# Mental Health, Academic Self-Efficacy and Study Progress Among College Students – The SHoT Study, Norway

**DOI:** 10.3389/fpsyg.2019.00045

**Published:** 2019-01-24

**Authors:** Kirsti Grøtan, Erik R. Sund, Ottar Bjerkeset

**Affiliations:** ^1^Faculty of Nursing and Health Sciences, Nord University, Levanger, Norway; ^2^HUNT Research Centre, Department of Public Health and Nursing, Faculty of Medicine and Health Sciences, Norwegian University of Science and Technology (NTNU), Levanger, Norway

**Keywords:** students, mental health, academic self-efficacy, study progress, help-seeking

## Abstract

Student life can be stressful and for some students it may cause mental distress. Besides being a major public health challenge, mental distress can influence academic achievement. The main objectives of the current study were to examine associations of mental distress with academic self-efficacy and study progress. A secondary aim was to examine mental health help seeking for students with mental distress. Data was derived from the Norwegian Students’ health and welfare survey 2014 (SHOT 2014) which is the first major survey comprising questions of both mental health, academic self-efficacy and psychosocial factors amongst students. Utilizing these data for a Norwegian region, we found that 749 (31%) of the 2430 Norwegian full-time students under the age of 35 responded to the survey. Symptoms of mental distress were measured using the Hopkins Symptom Checklist (HSCL-25) and academic self-efficacy was measured using a Norwegian version of the General Self-Efficacy Scale (GSE) tailored to the academic setting. Demographic-, social, lifestyle, and study-related variables were included in the analyses. Logistic regression analyses were performed to assess the relationship between mental distress, academic self-efficacy, and academic performance. Seventeen percent reported severe symptoms of psychological distress which is similar to the overall prevalence among students in Norway. Students reporting severe mental distress were four times as likely to report low academic self-efficacy and twice as likely to report delayed study progress compared to students reporting few or moderate symptoms of mental distress. 27% of those reporting severe mental distress had sought professional help whereas 31% had considered seeking help. The study showed that there was a strong association between symptoms of mental distress, academic self-efficacy and study progress. Prospective studies should evaluate whether improved help-seeking and psychological treatment can promote students mental health and ultimately improve academic self-efficacy and study progress.

## Introduction

Today’s younger generation represents the largest group of students in history. The transition from adolescence into young adulthood involves major changes in several areas – financial, housing, social, and emotional – and this transition period can cause relational challenges that some young adults experience as stressful. It has also been maintained that the proportion of students who experience their student life as mentally stressful is increasing (Nedregård and Olsen, 2014). This trend may suggest that students experience this period increasingly demanding, and for some of them it may be a direct cause of mental illness (Nerdrum et al., 2009).

Internationally, students’ mental health is highlighted as a major public health challenge (Stallmann, 2008; Storrie et al., 2010). A systematic review found that half of the students who reported mental distress symptoms also had experienced these symptoms before they began their studies, while the remaining half developed symptoms during their studies. Other studies, from the United States, Canada, and United Kingdom, confirm high(er) rates of mental health problems among university students, compared to the general population in the same age group (Adalf et al., 2001; Bewick et al., 2010; Keyes et al., 2012). Mental distress has been linked to lower academic self-efficacy and poor study progress, yet underpinning mechanisms are complex and not fully elucidated. A longitudinal study from the United States found that mental health problems predicted delayed academic success (GPA), thus suggesting a direction of influence (Eisenberg et al., 2009). Further, there may be factors associated with both these factors operating on a number of levels, from individual factors to interpersonal issues and institutional characteristics. Of individual level factors, previous studies have reported that emotional problems had a negative effect on study progress and on the dropout rate from higher education (Robbins et al., 2004; Storrie et al., 2010).

Internationally, and particularly in the United States, a significant amount of research on the transition to higher education has been carried out over the last 40 years. This has contributed to development of a broader theoretical framework for understanding the factors important for college success. There are mainly two directions that points out this work; the sociological theories of education, such as Astin (1993) and Tinto (1993), and social cognitive learning theory by Bandura (1997) and Pascarella and Terenzini (2005). Astin emphasizes the importance of students taking part in the learning environment (Astin, 1993). Tinto further developed Astin’s theories by emphasizing students’ own driving forces as motivation, intentions, and adherence to education (Tinto, 1993).

Both anxiety and depression are detrimental to academic and social participation in everyday student life (Byrd and McKinney, 2012; Keyes et al., 2012; Salzer, 2012). Depressive disorders result in lowered mood, reduced cognitive function, lack of a sense of coping and interest in others, as well as lack of energy (Mykletun et al., 2009). In turn, depression and anxiety often affect memory and concentration, which makes it more difficult to acquire new knowledge and cope with examination situations. This will often reinforce perceptions of hopelessness and inadequacy, and in many people it will sustain the feeling of anxiety and depressed mood in a vicious circle (Rice et al., 2006; Stallmann, 2008). On the other hand, and depending on the symptom level, some uncertainty and anxiety in the academic situation may contribute to increased work effort and possibly improved results (Andrews and Wilding, 2004; Nedregård and Olsen, 2014).

The concept of self-efficacy refers to individuals’ own beliefs about capabilities to organize and execute the courses of action required to produce given attainments (Bandura, 1997). In educational psychology research self-efficacy has been shown to predict Student’s academic performance and progress across academic areas and levels (Pajares and Schunk, 2006; Vuong et al., 2010). Academic self-efficacy has been proven to be a powerful predictor when the critical performance is as global as the self-efficacy level measured (Choi, 2005; Zajacova et al., 2005). In studies of academic performance and persistence, social cognitive theory has proved to serve as a well-suited model (Brown et al., 2008).

Bandura’s social cognitive learning theory emphasizes the inherent ability to develop control over thoughts, feelings, and actions. This approach focuses on cognitive processes in individual adaptation and interaction with the social environment, suggesting that poor social mastering reduces the capacity to build supportive social relationships (Bandura, 1997). A central concept is self-efficacy describing the individual’s belief in their own coping in different situations. Low self-efficacy affects both achievements, ambitions, and motivation (Bandura, 1986; Dinther et al., 2011). Further, Bandura (1997) also linked experiences of persistent overthinking and negative self-esteem to the development of symptoms of anxiety and depression.

Also, students’ ability to handle emotional stress during their studies was found to be an important factor in preventing academic delay and dropout (Storrie et al., 2010). In a review article based on studies among students in Australia, the authors reported that loneliness, also commonly linked to depression, was an independent risk factor for low study progress (Heinrich and Gullone, 2006). Other studies have reported that students have a number of concerns with their studies, with expectations about performing, and also report financial insecurities (Stewart-Brown et al., 2000; Stallmann, 2008).

The previous Students’ Health and Welfare Survey in 2010 (SHoT 2010) showed that 25% of students in Norway reported moderate or severe symptoms of mental health problems and 13% reported severe symptoms relating to mental health problems (Nedregård and Olsen, 2010). This is considerably higher than the rest of the population, where 12% in the same age group reported moderate or severe symptoms (Amdam and Vrålstad, 2012).

Compared to the general population and workforce, available knowledge about students’ mental health is scarce. There is also insufficient knowledge about how mental health influences, or is influenced by academic progress. In addition, although Norwegian population based studies (Tyssen et al., 2004) and international student surveys (Zivin et al., 2009; Verouden et al., 2010; Eisenberg et al., 2012) report low mental health help seeking, we know little about the extent to which Norwegian students seek and receive appropriate mental health care.

We therefore examined the following research questions among college students participating in the SHoT study:

(1) How many of the students at a College in Norway report having severe mental health problems?
(2) Do students who report severe symptoms of mental health problems have a higher risk of low academic efficacy and poor study progress compared to students reporting few and moderate symptoms?
(3) To what extent do students at this College seek help for mental health problems?

## Materials and Methods

### Study Sample and Setting

The Students’ Health and Welfare Survey (SHoT) is a cross-sectional questionnaire survey for Norwegian full-time students under the age of 35, conducted in February 2014 (Nedregård and Olsen, 2014). Students taking single courses and credit points that do not lead to a specific degree were excluded from the study. International students were also excluded from the study because previous SHoT studies in Trondheim and Oslo have shown that they are a heterogeneous group with challenges that differ considerably from those faced by Norwegian residents and/or citizens who are students (Nedregård and Olsen, 2014). Of all eligible students nationwide, a randomly drawn 10% sample were invited to SHoT. The only exception was this College, where all students in the total sample were invited. This decision was made by the student welfare association connected to the College to ensure that the sample was large enough to make it possible to process and interpret the data for this institution. Of a total of 20 invited Student Welfare Associations, 10 participated in SHoT 2014, and together they represent 71% of all students in the target group (Nedregård and Olsen, 2014). Of a total of 47,514 students invited students nationally, 13,663 participated (29%). At this College, 2,430 were invited, and 749 (31%) participated. The share of women among participants was 69% and the corresponding figure among the invited was 62%. With respect to age, 78.2% among the invited and 78.6% of the participants were in the age group 18–25.

### Data Collection

An email with a link to the online questionnaire in Questback was sent to all students in the sample.

Most educational institutions gave access to both personal and student email addresses. At this University College, though, only student email addresses were used. In addition to the questionnaire, a cover letter was sent, informing the students that participation was voluntary and, among other details, that it was possible to leave some questions out. The link to the questionnaire was designed in a fashion that made it possible to save and return to the questionnaire several times, so no one felt pressured to complete it in one sitting. Data collection was conducted from 24th of February 2014 to 27th of March 2014. The Student Welfare Association helped market the survey using information material developed by the Communication department of the Student Welfare Associations. Information about SHoT was provided on the Student Welfare Association website and Facebook page, via student media, the University College’s Facebook page, flyers and information stands at all campuses. To optimize the response rate, the Student Welfare Association offered a tablet computer and 10 gift certificates worth a total of NOK 3,000 as prizes for use in the student association’s facilities.

The formal agreement between the conductors and The Student Welfare Association described how personal data were processed, and all data were processed according to Norway’s Personal Data Act.

### The Questionnaire in SHoT

The questionnaire for SHoT 2014 is a revised version of the form used in SHoT 2010, which in turn was based on previous health and well-being studies among students in Oslo (HELT 2003 and 2005) and Trondheim (HOT 2004 and 2007) (Nedregård and Olsen, 2014).

The survey consists of 66 questions and instruments that assess the student’s health and well-being; financial situation, housing, family situation, lifestyle, issues specific to studying as well as physical and mental health were all charted in the survey – with a particular emphasis on psychosocial issues. The conductors of the National survey designed the questionnaire in cooperation with the steering committee for SHoT, which included representatives from the Student Welfare Associations in Trondheim, Bergen, and Oslo (Nedregård and Olsen, 2014).

### Measurement Instruments

#### HSCL-25 (Hopkins Symptom Checklist-25)

Hopkins Symptom Checklist-25 is a widely used self-report instrument which measures several underlying dimensions of psychological distress, including anxiety and depression (Derogatis et al., 1974). In short, the HSCL-25 scale consists of two main subscales: a 10-item anxiety symptom scale and a 15-item depressive symptom Likert scales (Winokur et al., 1984; Tambs and Moum, 1993). Participants are asked to assess the subjective anxiety and depression symptom load in the past 2 weeks, and response categories were ‘Not at all,’ ‘A little,’ ‘Quite a bit,’ or ‘Extremely.’ Importantly, neither HSCL-25 nor other self-report instruments alone can be used to diagnose mental illness.

An average score above 1.75 indicates moderate to severe symptom load in the two last weeks and is often used as the cut-off point in scientific studies (Winokur et al., 1984; Tambs and Moum, 1993). In this study, however, a cut-off point at 2.0 is used and indicates a severe symptom load (Nedregård and Olsen, 2014).

To date, HSCL-25 has been utilized across various populations and settings; in psychiatric patients (Veijola et al., 2003), in the general population (Nyman et al., 2010), and in immigrants and minority groups (Mouanoutoua and Brown, 1995; Hoffmann et al., 2006). The psychometric properties of the HSCL-25 indicate adequate reliability and sensitivity of the subscales (Deane et al., 1992).

#### GSE (General Self-Efficacy Scale)

In SHoT 2014, academic self-efficacy was measured using a Norwegian version of the General Self-Efficacy Scale (GSE) (Jerusalem and Schwartzer, 1992), especially tailored to the academic setting. The GSE was adapted so that all the statements evaluate one’s own efficacy as a student, rather than general trait self-efficacy (Nedregård and Olsen, 2014).

The GSE is based on the concept of self-efficacy derived from social cognitive learning theory, developed by Bandura (1997). The concept describes individuals’ confidence in their own ability to cope with stress and various challenges.

The instrument consists of 10 statements on a four-point Likert-scale ranging from 1 *(“completely wrong*”) to 4 (“*completely correct*”). Examples of statements included in the GSE:

‘I can always manage to solve difficult problems in my studies if I try hard enough’

‘If someone opposes me at school, I can find the means and ways to get what I want’

‘It is easy for me to stick to my study aims and accomplish my goals.’

Average scores above 3.5 are defined as indicating high self-efficacy, scores between 2.5 and 3.5 are defined as average self-efficacy and scores under 2.5 as low self-efficacy (Nedregård and Olsen, 2014).

Results suggest that academic self-efficacy beliefs predict college outcomes but that this relationship is dependent on when efficacy beliefs are measured, the types of efficacy beliefs measured, and the nature of the criteria used (Gore, 2006).

### Study Progress

In SHoT, the students were asked about their study progression in relation to nominal study length: *‘*Do you currently follow the nominal study progress in relation to nominal study length in the current semester (30 credits in one semester)?’ *Response* options were: ‘delayed,’ ‘not delayed,’ or ‘don’t know.’

Of note, first semester students were excluded in the analyses of this question.

### Ethics Statement

An invitation with a link to the questionnaire was sent out, and a cover letter was attached explaining the purpose of the study; participation was voluntary and participants could skip some of the questions if they did not want to answer them. All participants were adults and their consents were obtained by virtue of survey completion. The invitation also stated that data from the survey would be anonymized by the conductors of the survey, the email addresses would be deleted after the last reminders had been sent out and the winners of the incentives had been drawn. Those invited were also informed that the conductors of the survey had received approval from the Norwegian Centre for Research Data (NSD) to process the data and to anonymize them in accordance with Section 7-27 of the Personal Data Regulations, reported to NSD on 15 January 2014, case number 37102 (Nedregård and Olsen, 2014). This included access to, and analyses of anonymized data only. As part of the standard procedure, all e-mail addresses were deleted after the response, and before the database was established.

Hence, it was not necessary to obtain any additional written confirmed consent in this study. In cases where a written confirmed consent is required, NSD will refer the researcher/applicant to the Regional Committees for Medical and Health Research Ethics (REC), Norway^1^.

As a student counselor employed at the Student Welfare Association, the first author was actively involved in the information and recruitment campaign prior to SHoT 2014, at several campuses. It is uncertain if this may have affected participation, but there is little reason to believe that it has influenced the way that students answered the individual questions. The possibility to win prizes may also have influenced the students’ willingness to participate in the investigation.

### Statistical Analysis

Logistic regression models were specified whereby low academic efficacy and delayed academic performance were regressed on symptoms of mental distress in separate analyses (model 1). Subsequent models were built sequentially by adding likely confounding factors and finally adjusted for all (model 5). We did a complete case analysis and made no attempt to impute missing values on covariates. In the analysis of delayed academic performance, we discarded first-semester students (*N* = 79) since it was simply not possible for them to be delayed in their study progression at the time of survey. Excluded were also those who responded “don’t know” on this question (*N* = 88). The net samples were thus 659 and 523 for low academic efficacy and delayed academic performance, respectively. We report odds ratios (OR) as our effect measure along with 95% confidence intervals (95% CI). Analyses were conducted in SPSS (v 22).

## Results

In SHoT 2014, a total of 2,430 full-time students at the College under the age of 35 were invited. Of these, 749 (31%) answered the survey. Most of the respondents were women; in the total National sample 62.1% were women and at this College they accounted for 68.9% of participants (Nedregård and Olsen, 2014). Among the 749 participants at the College, 32% reported symptoms of mental health problems of moderate or severe degree (cut-off 1.75 points) and 17% reported symptoms of severe mental health problems (cut-off 2.0 points) during the past 2 weeks (research question 1). In further analyses, 2.0 was used as the cut-off point for symptoms of mental health problems in this study.

Table 1 shows that almost half the participants were in the age group 18–25. In terms of marital/cohabitation status, 36% lived with spouse/partner, 18% were in a relationship yet lived alone, and 45% were single. Overall, 25% lived alone. Only 13% of the participants had children in their care, while more than 60% described themselves as financially vulnerable. Nearly 13% regarded themselves as lonely (socially and emotionally). As few as 7% stated that they smoked everyday, and about 6% drank alcohol more than twice per week. One in three (32%) described themselves as physically inactive, and the same proportion spent 0–19 h per week on their studies.

**Table 1:** Sample characteristics, the SHoT survey (*N* = 749).

	*N*	%
**Demographic and social**		
Age group		
18–20	154	20.6
21–22	232	31
23–25	203	27.1
26–28	83	11.1
29+	77	10.3
Gender		
Female	516	68.9
Male	233	31.1
Marital status		
Married/partner/cohabitant	273	36.4
Romantic partner	136	18.2
Single	334	44.6
*Missing*	*6*	*0.6*
Living alone		
Yes	184	24.6
No	565	75.4
Caring for children		
Yes	96	12.8
No	652	87
*Missing*	*1*	*0.1*
Financially vulnerable		
Yes	294	60.5
No	453	39.3
*Missing*	*2*	*0.3*
Loneliness (social and emotional)		
Yes	93	12.4
No	643	85.8
*Missing*	*13*	*1.7*
Lifestyle		
Daily smoker		
Yes	36	4.8
No	693	92.5
*Missing*	*20*	*2.7*
Alcohol use		
> = 2 times per week	46	6.1
<2 times per week	684	91.3
*Missing*	*19*	*2.5*
Physical activity		
Inactive	240	32
Active	480	64.1
*Missing*	*29*	*3.9*
**Academically related**		
Time spent on study (per week)		
Over 40 h	186	24.8
20–39 h	277	37
0–19 h	246	32.8
*Missing*	*40*	*5.3*
Stage of study		
1st semester	79	10.5
2nd–3rd semester	247	33
4th–5th semester	198	26.4
6th–8th semester	172	23
9th+ semester	41	5.5
*Missing*	*12*	*1.6*

Table 2 shows that female students reported severe symptoms of mental health problems twice as frequently as male students, 20 and 10% respectively. In total, 14% reported low academic self-efficacy and 6% reported delayed study progress.

**Table 2:** Descriptives of outcomes and main predictor of interest, by gender.

	Females		Males		Total	
	*N*	%	*N*	%	*N*	%
**Outcomes**						
Academic self-efficacy						
Low	75	14.6	29	12.4	104	13.9
Medium/high	429	83.1	199	85.4	628	83.8
*Missing*	*12*	*2.3*	*5*	*2.2*	17	2.3
Followed scheduled study progress						
No	30	5.8	16	6.9	46	6.1
Yes	396	76.7	188	80.7	584	78.0
Don’t know	90	17.4	29	12.5	119	15.9
**Main predictor of interest**						
Symptoms of mental distress (HSCL)						
Severe	104	20.2	22	9.4	126	16.8
Few or moderate	408	79.1	209	89.7	617	82.4
*Missing*	*4*	*0.8*	*2*	*0.9*	6	0.8

Table 3 shows bivariate relationships between various independent variables and the two dependent variables: low academic self-efficacy and delayed study progress. We found that the risk of experiencing low academic self-efficacy was more than four times higher [OR 4.55 (95% CI 2.79–7.42)] among those who reported symptoms of severe mental health problems than among those who reported few and moderate symptoms.

**Table 3:** Bivariate associations between various predictors and respective low academic self-efficacy and delayed study progress.

	Low academic self-efficacy		Delayed study progress	
	OR	95% CI	OR	95% CI
Symptoms of anxiety and depression (HSCL-25)				
Few or moderate	1	Ref.	1	Ref.
Severe	4.55	(2.79–7.42)	2.47	(1.19–5.13)
Age groups				
18–22	1	Ref.	1	Ref.
23–25	0.96	(0.57–1.64)	1.58	(0.70–3.56)
26+	0.92	(0.52–1.62)	1.83	(0.81–4.15)
Gender				
Female	1	Ref.	1	Ref.
Male	0.76	(0.46–1.26)	1.1	(0.55–2.22)
Marital status				
Married/partner/	1	Ref.	1	Ref.
cohabitant
Romantic partner	0.59	(0.29–1.20)	0.81	(0.28–2.36)
Single	1.00	(0.62–1.61)	1.22	(0.59–2.54)
Living alone				
No	1	Ref.	1	Ref.
Yes	1.02	(0.61–1.72)	1.24	(0.58–2.63)
Care for children				
No	1	Ref.	1	Ref.
Yes	1.27	(0.68–2.37)	1.05	(0.39–2.79)
Financially vulnerable				
No	1	Ref.	1	Ref.
Yes	1.85	(1.18–2.90)	1.06	(0.54–2.10)
Loneliness (social and emotional)				
No	1	Ref.	1	Ref.
Yes	2.6	(1.50–4.49)	1.96	(0.86–4.48)
Physical activity				
Active	1	Ref.	1	Ref.
Inactive	1.26	(0.80–2.01)	1.55	(0.79–3.06)
Time spent on study (per week)				
Over 40 h	1	Ref.	1	Ref.
20–39 h	0.48	(0.26–0.87)	0.77	(0.35–1.70)
0–19 h	1.22	(0.72–2.08)	0.69	(0.29–1.65)
Stage of study				
1st semester	1.41	(0.66–3.02)		
2nd–3rd semester	1	Ref.	1	Ref.
4th–5th semester	1.39	(0.78–2.49)	0.27	(0.08–0.99)
6th–8th semester	1.17	(0.64–2.14)	1.43	(0.64–3.20)
9th+ semester	0.64	(0.18–2.21)	3.71	(1.40–9.84)
	*N* = 659		*N* = 523	

*Odds ratio (OR) and 95% confidence interval (95% CI).*

Among students who reported loneliness (social and emotional), the odds of low academic self-efficacy were approximately 2.6 times higher [OR 2.6 (95% CI 1.50–4.49)] than for those who did not report loneliness. Those who reported financial vulnerability had almost twice the risk [OR 1.85 (95% CI 1.18–2.90)] of delayed study progress compared with the reference group (not financially vulnerable). Students who spent 20–30 h per week on their studies had a significantly lower risk [OR 0.48 (95% CI 0.26–0.87)] while those who spent 0–19 h per week had a higher risk [OR 1.22 (95% CI 0.72–2.08)] of reporting low academic self-efficacy compared with those who spent more than 40 h on their studies per week.

The odds of reporting delayed study progress was more than twofold increased [OR 2.47 (95% CI 1.19–5.13)] for those with symptoms of severe mental health problems compared with those who reported few and moderate symptoms. The analyses also show a strong association between delayed study progress and loneliness, living alone, and physical inactivity, yet we found no sound statistical evidence for this.

Gender, age, marital status, living alone, and caring for children showed some association with both low academic self-efficacy and delayed study progress, but these were not statistically significant either (all *p*-values > 0.05).

For research question 2, the multivariable regression analyses (Table 4) confirmed that symptoms of severe mental health problems were strongly associated with low academic self-efficacy; the crude odds ratio was robust to stepwise adjustment, and the final model (Model 5) still suggests a near fourfold increased risk [OR 3.82 (95% CI 2.25–6.49)].

**Table 4:** The risk of low academic self-efficacy among students.

	Model 1		Model 2		Model 3		Model 4		Model 5	
	OR	95% CI	OR	95% CI	OR	95% CI	OR	95% CI	OR	95% CI
Symptoms of anxiety and depression (HSCL-25)										
Few or moderate	1	Ref.	1	Ref.	1	Ref.	1	Ref.	1	Ref.
Severe	4.55	(2.79–7.42)	4.01	(2.40–6.69)	4.28	(2.61–7.01)	4.72	(2.87–7.79)	3.82	(2.25–6.49)
Social/emotional loneliness										
No			1	Ref.					1	Ref.
Yes			1.67	(0.92–3.02)					1.83	(0.99–3.38)
Financially vulnerable										
No					1	Ref.			1	Ref.
Yes					1.60	(1.00–2.55)			1.75	(1.08–2.83)
Hours spent on studies per week										
More than 40							1	Ref.	1	Ref.
20–39							0.50	(0.27–0.93)	0.51	(0.27–0.95)
0–19							1.38	(0.79–2.40)	1.51	(0.86–2.66)
Log likelihood	−243.80		−242.45		−241.86		−237.42		−233.21	

*Odds ratio (OR) and 95% confidence interval (95% CI). N = 659.*

The variables loneliness, financial vulnerability and hours spent studying also showed a strong degree of association and were thus appropriate for inclusion in the multivariable analyses regarding low academic self-efficacy. Although the association between loneliness, living alone, physical inactivity and delayed study progress were not statistically significant, they were considered relevant for further inclusion in the analyses. The variables gender and age showed no statistically significant association with low academic self-efficacy and delayed study progress and they had no effect on the association between symptoms of mental health problems and the two outcomes. Table 5 indicates similar patterns for “delayed study progress”; the final model still showed a twofold increase in risk [OR 2.14 (95% CI 0.97–4.72)] for students reporting severe psychological symptom levels, compared to mild or moderate levels (research question 2).

**Table 5:** The risk of delayed study progress among students.

	Model 1		Model 2		Model 3		Model 4		Model 5	
	OR	95% CI	OR	95% CI	OR	95% CI	OR	95% CI	OR	95% CI
Symptoms of anxiety and depression (HSCL-25)										
Few or moderate	1	Ref.	1	Ref.	1	Ref.	1	Ref.	1	Ref.
Severe	2.47	(1.19–5.13)	2.22	(1.01–4.86)	2.46	(1.19–5.11)	2.35	(1.12–4.91)	2.14	(0.97–4.72)
Social/emotional loneliness										
No			1	Ref.					1	Ref.
Yes			1.44	(0.59–3.51)					1.35	(0.55–3.35)
Living alone										
No					1	Ref.			1	Ref.
Yes					1.21	(0.57–2.60)			1.19	(0.56–2.56)
Physical activity										
Active							1	Ref.	1	Ref.
Inactive							1.41	(0.71–2.80)	1.36	(0.68–2.73)
Log likelihood	−130.98		−130.68		−130.86		−130.52		−130.20	

*Odds ratio (OR) and 95% confidence interval (95% CI). N = 523.*

Addressing research question 3, Table 6 shows that one in four students with symptoms of severe mental health problems had sought help for these problems during the past 12 months. In the group with few and moderate problems, approximately 7% had sought help for psychological problems. In absolute numbers, however, more students have sought help in the group that reported few and moderate mental health problems than in the group reporting severe mental health problems. The analysis also revealed that in each group about the same number had not sought help but had considered doing so.

**Table 6:** Help-seeking behavior and symptoms of mental distress by gender.

	Few/moderate symptoms						Severe symptoms						
	Females		Males		Total		Females		Males		Total		
	*N*	%	*N*	%	*N*	%	*N*	%	*N*	%	*N*	%	Grand Total
Yes	35	8.8	6	2.9	41	6.8	32	28.8	4	18.2	36	27.1	77(10.5%)
No, but considered	42	10.6	11	5.3	53	8.8	31	27.9	10	45.4	41	30.8	94(12.8%)
No	319	80.6	190	91.8	509	84.4	48	43.3	8	36.4	56	42.1	565(76.9%)
Total	396	100	207	100	603	100	111	100	22	100	133	100	736(100%)

Overall, Table 6 confirms that men seek help for psychological problems to a lesser degree than women.

## Discussion

In the national SHoT survey for Norwegian colleges and universities in 2014 we examined the prevalence of mental health problems, their influence on academic self-efficacy and study progress, as well as self-reported help seeking for mental health problems. Overall, 17% of full-time students at this particular College reported symptoms of severe mental health problems, 14% reported low academic self-efficacy and 6% reported delayed study progress. Of those with symptoms of severe mental health problems, 27% had sought help for mental health problems, and 31% reported that they had considered this, but had not yet done so. Further, students who reported symptoms of severe mental health problems had four times the risk of low academic self-efficacy and twice the risk of delayed study progress, compared with those who reported few and moderate symptoms of mental health problems.

### Prevalence of Severe Mental Health Problems Among Students

The prevalence of moderate and severe mental health problems among the students proved to be at about the same level as for the total sample in the national SHoT survey in 2014 (Nedregård and Olsen, 2014). Compared with the general Norwegian population in the same age group, using the same instruments, students report a twofold increased prevalence of symptoms of moderate and severe mental health problems (25 vs. 12%, cut-off at 1.75) (Amdam and Vrålstad, 2012). This finding may indicate that there is something about academia and the study situation that makes students experience their study years as stressful, and for some this triggers mental illness (Adalf et al., 2001; Nerdrum et al., 2009).

The national results from SHoT 2014 also show that the total number of students reporting symptoms of severe mental health problems has increased compared with numbers from SHoT 2010 (Nedregård and Olsen, 2010). This is consistent with the overall trend among young adults in Norway from 2008 to 2012. In both studies, this is primarily explained by increased prevalence among women (Amdam and Vrålstad, 2012; Nedregård and Olsen, 2014).

In line with our findings, some international studies, where the same instruments have been used, confirm that students report mental health problems more often than non-students in the same age group (Roberts et al., 1999; Adalf et al., 2001; Stallmann, 2008; Ibrahim et al., 2013). At the same time, there are some major methodological differences between these studies, making direct comparison of results difficult. Further, samples sizes are all too often insufficient to establish sound statistical evidence (Cook et al., 2006).

Furthermore, we found that almost 13% of the students described themselves as lonely (socially and emotionally). The transition from living with family and having a social network that has been built up over time, to settling into new places, establish new friendships and integrating into new social communities, may be difficult for some students (Nerdrum et al., 2009; Eisenberg et al., 2012). In addition, the figures show that over 60% of the students describe themselves as financially vulnerable, which is in line with previous studies (Nerdrum et al., 2009; Eisenberg et al., 2013).

### Student Mental Health, Academic Self-Efficacy and Study Progress

Our study indicates that students who report symptoms of severe mental health problems have about four times the risk of experiencing low academic self-efficacy compared with those who report few and moderate symptoms of mental health problems. A situation in which anxiety contributes to worries, motor restlessness, unfounded fear of not accomplishing things, in combination with procrastination and avoidance behavior, may contribute to students developing problems in participating actively in learning and study situations. This may in turn contribute to avoidance, isolation and loneliness, leading to poorer academic- and social inclusion with both fellow students and staff at the educational institution (Byrd and McKinney, 2012; Salzer, 2012). Our study shows that there is an association between mental health problems and academic self-efficacy, but we cannot make any claims about the causal direction. In exploring the relationship between psychosocial factors, study skills, and academic outcomes, the authors of a review study from the United States point out the lack of empirical studies that combine sociological education research and psychological theory in higher education (Robbins et al., 2004).

Tinto’s research points to commitment as a particularly important factor in academic performance, best developed through professional and social participation facilitated by the educational institutions (Tinto, 2006). Students’ experiences in the learning processes might give educational institutions a clearer picture of the factors that contribute to motivation and persistence (ibid; Reason et al., 2006).

In our study, students who reported symptoms of severe mental health problems were twice as likely to report delayed study progress compared with those who reported few and moderate mental health problems. However, there is little knowledge about the relationship between psychological distress and completion of credits among Norwegian students (Hovdhaugen and Aamodt, 2009). A review article that included 11 studies from three different countries, however, confirmed a clear association between poor emotional health and delayed study progress (Storrie et al., 2010). In addition, several international studies report a strong association between self-efficacy and academic performance (Robbins et al., 2004; Fenollar et al., 2007; Richardson et al., 2012).

In keeping with the literature, the authors of a Swedish longitudinal study reported a weak association between mental health and degree completion (Vaez and Laflamme, 2008). They found that low academic self-efficacy had a clear association with low completion of credits. In contrast, a cross-sectional study in the United States, in which clinical instruments were used to investigate the relationship between psychiatric diagnoses and academic performance, researchers found that generalized anxiety had a positive influence on academic performance (Svanum and Zody, 2001; Pritchard and Wilson, 2003). The fact that academic self-efficacy has been identified as a mediator between anxiety and academic performance, may help explain some contradictory results in different studies. Overall, though, they reported that mental health issues showed a weak, negative association with academic performance (Svanum and Zody, 2001). Further, they suggest that students’ ability to cope with the academic situation depends, at least partly, on their understanding of and ability to handle emotional difficulties (Svanum and Zody, 2001). A meta-analytic investigation among Community College Students in the United States reported that anxiety seemed unrelated to both study persistence and college achievement, and that stress can impact both positively and negatively on students’ achievements (Fong et al., 2017).

### Mental Health Help Seeking in College Students

We found that 27% of the students with symptoms of severe mental health problems had sought help, while 31% had considered seeking help. Both Norwegian and international studies confirm that many students refrain from seeking help for psychological problems (Tyssen et al., 2004; Zivin et al., 2009; Verouden et al., 2010; Eisenberg et al., 2012). This is in keeping with evidence from the general population, which shows that low help seeking and under-treatment of anxiety and depression is pervasive and should receive more attention (Kessler et al., 2005; Roness et al., 2005).

The reasons students refrain from seeking help may be many and varied. A barrier to seeking help for psychological difficulties is that students want to be “normal” and not to stand out from the crowd, while others have the opinion that stress and difficulties are normal parts of a student’s life (Verouden et al., 2010). Increased knowledge about support services on campus and interventions that reduce stigma would probably contribute to more students seeking help (Quinn et al., 2009; Storrie et al., 2010; Reavley and Jorm, 2010; Eisenberg et al., 2012). A review article based on studies mainly from the United States concludes that an effective way to reach students who need help, would be to offer interventions to a larger group of students (Regehr et al., 2013).

Educational institutions must have productive collaborative relationships, anchored in the whole institution, with counseling and health services both on campus and in the public health service to make it possible to offer effective help to students with serious mental health problems (Stanley and Manthorpe, 2001). Preventive initiatives at various levels and in various settings can raise the awareness of those who need help with their own problems and inform students about the support services that are available (Hunt and Eisenberg, 2010). Closer collaboration between the Student Welfare Association’s counseling services, the educational institution and the public health service could also help increase the number of students who seek and are offered help, which in turn contribute to a better learning environment for all students (Storrie et al., 2010; Eisenberg et al., 2012).

Given the prevailing focus on the relationship between mental health, learning environment, social and academic affiliation and dropout from secondary education, it is paradoxical that this thinking receives little attention as a research question within higher education in Norway.

Among the help seekers at this college, only 23% had sought help from the Student Welfare Association’s counseling services. In contrast, the three largest Norwegian universities located in Bergen, Trondheim and Oslo have the highest proportion of students seeking help from the counseling and health services of the Student Welfare Association, 58, 53, and 45% respectively (not shown in Results). Importantly, an overview of the Student Welfare Association’s health and counseling services shows that these universities offer a wider range of services with greater scope (including psychologists) than the smaller Student Welfare Associations (Ministry of Health and Care Services, 2008). Overall, the resources available for counseling and health services vary considerably across campuses, and this is true both in Norway and internationally (Hunt and Eisenberg, 2010; Nedregård and Olsen, 2014).

### Strengths and Limitations

This study has several strengths. SHoT 2014 is a survey with a wide scope and a high number of participants. The total database includes responses from 13,663 students (Nedregård and Olsen, 2014). The questionnaire in SHoT 2014 is a revised version of the one used in 2010, and mainly validated instruments have been used in the design of the form. In addition, there is little missing data among the responses. For the variable regarding symptoms of mental health problems, 6 respondents did not answer the question and for academic self-efficacy and study progress, 59 did not answer the question.

At the same time, some limitations must be kept in mind in the interpretation of the results. A response rate of 31% in a questionnaire survey is usually regarded as low, which increases the risk of systematic bias (Ringdal, 2007). According to the conductors of SHoT, students’ response rate to questionnaires is lower than the rate for other groups (Nedregård and Olsen, 2014). In Internet-based surveys conducted among students, it is therefore not unusual for the response rate to be around 25% (Nedregård and Olsen, 2014). Fortunately, those who answered are relatively representative in terms of gender and age distribution compared to the drawn total sample. In addition, this is a cross-sectional study, and we cannot draw conclusions about cause-effect mechanisms (Ringdal, 2007). In simple terms, symptoms of severe mental health problems may *lead to* low academic self-efficacy, but they can also be a *consequence of* low academic self-efficacy. The same applies to the association between symptoms of severe mental health problems and poor study progress.

## Conclusion

This study confirms previous findings regarding the relatively high occurrence of mental problems among students and low levels of help-seeking (Tyssen et al., 2004; Zivin et al., 2009; Verouden et al., 2010; Eisenberg et al., 2012). The strong association between psychological distress and academic self-efficacy has also been described in previous studies (Karademas and Kalantzi-Azizi, 2004; Byrd and McKinney, 2012), yet the causal pathways and underpinning mechanisms are not fully understood.

First, we suggest that future studies should assess students’ health prior to study start and follow them up with repeated measurements and qualitative interviews. This methodology could provide useful knowledge about mental health problems and how they arise and influence academic self-efficacy and study progress. Second, counseling and health services should be easily available and offered on campus, to facilitate and increase mental health literacy and help seeking – both represent key challenges across societal and health care settings. More specifically, psychoeducational interventions, counseling, guidance and treatment, group initiatives, stigma reducing, and health-promoting measures could be carried out and evaluated in the university setting. Finally, research that combines educational and psychological theory in higher education might contribute further to a more complete understanding of the associations between mental health, academic self-efficacy and study progress.

## Author Contributions

KG, ES, and OB contributed substantially to the conceptualisation and design of the study. KG and ES performed the statistical analysis. KG wrote the first draft of the article. ES and OB revised it critically for important intellectual content. All authors read and approved the final version of the manuscript and contributed to the interpretation of the data.

## Conflict of Interest Statement

The authors declare that the research was conducted in the absence of any commercial or financial relationships that could be construed as a potential conflict of interest.

## Footnotes

1 https://helseforskning.etikkom.no/forside?_ikbLanguageCode=us
